# Web-based sensitivity training for interacting with facial paralysis

**DOI:** 10.1371/journal.pone.0261157

**Published:** 2022-01-21

**Authors:** Nicole Zhang, Kathleen Bogart, John Michael, Luke McEllin

**Affiliations:** 1 Department of Psychology, Warwick University, Coventry, United Kingdom; 2 School of Psychological Science, Oregon State University, Corvallis, Oregon, United States of America; 3 Department of Cognitive Science, Central European University, Vienna, Austria; National Institutes of Health, UNITED STATES

## Abstract

Previous research has shown that observers tend to form inaccurate and negatively biased first impressions of people with facial paralysis (FP). It has been hypothesised that this may be ameliorated by encouraging people to focus on channels of expression other than the face. This was tested in a web-based study of 466 participants. Participants in the Trained Condition received tips for perceiving expressiveness in individuals with FP, while those in the Untrained Condition received general medical information about FP. We observed no significant differences between groups for accuracy of emotion recognition, but a significant effect of the training upon perception of emotional intensity. These results show that attending to non-facial cues may improve social perception and reduce bias.

## Introduction

The human face is a primary channel for expressing emotions and for communicating subtle cues in social interaction [1]. These important social functions of facial expression can be impaired in cases of weakness or paralysis of the facial musculature. Facial paralysis (FP)—which affects approximately 100,000 people in the UK [2]—is mainly caused by temporary or permanent damage to the facial nerve, which can be due to a range of causes, including Bell’s Palsy and Moebius Syndrome [1]. For example, Moebius Syndrome is a rare type of FP which occurs at birth as a result of damage to the 6th (abducens) and 7th (facial) nerves [3, 4].

### Background research

Given the importance of the face for expressing emotions and other mental states, and for conveying other non-verbal social cues, it is no surprise that some people with FP experience difficulties during social interactions: For instance, it has been demonstrated that individuals with facial paralysis report high levels of psychological distress [5] and poor social functioning [6]. On the other hand, many individuals with FP do not report difficulties with social interaction. This may be due in part to their adoption of alternative expressive strategies (e.g. gestures, body language and prosody) to compensate for their lack of facial expressiveness [7–10]. And indeed, some research has shown that interventions designed to encourage the use of such alternative compensatory strategies can help people with FP to build rapport in social interactions. For example, one study [11] tested the efficacy of an intervention designed to train teenagers with Möbius Syndrome (MS) to increase the use of alternative communication strategies (e.g. gestures) to compensate for their lack of facial expressivity. The results showed that observer-coded rapport was greater after the intervention, and that observer-coded gesture and expressivity increased in participants with and without MS. Fidgeting and repetitiveness of verbal behavior also decreased in both groups–especially in the group without MS. In sum, the intervention impacted nonverbal and verbal behavior in participants with and without MS, increasing rapport as well as overall gesturing. This is important because it indicates that some of the difficulties experienced by people with FP in social interactions arise from others’ discomfort or uncertainty. Insofar as this is correct, it may be beneficial to develop a social skills workshop for people–particularly for teachers and medical professionals–who do not themselves have FP but who are likely to interact with individuals who do have FP.

Indeed, research has also shown that lay people as well as healthcare providers tend to form inaccurate first impressions of people with dampened facial expression [12–14]. This may be due in part to limits which facial paralysis imposes upon the expression of information about emotional states [10]–i.e. since people are accustomed to receiving information about others’ mental states from their facial expressions, the absence of this expected information may cause an interaction partner to feel uncomfortable or confused about what the person with MS is thinking or feeling. This conjecture is corroborated by evidence that individuals with facial movement disorders such as MS or Parkinson’s disease are often perceived as unhappy, unfriendly, depressed, disinterested, or unintelligent [12, 14–16], making others less likely to pursue engagement and friendships with them [13]. In sum, the social difficulties experienced by many people with MS may lie partially with their interaction partners without MS, who may for various reasons feel uncomfortable or confused. To address this, Bogart & Tickle-Degnen [16] developed a brief training designed to encourage people who will encounter individuals with FP to focus on other channels of expression rather than the face, e.g. hand gestures, body language, tone of voice and speech content. Specifically, participants were randomly assigned to one of two training conditions or an untrained control. The Education Condition was designed to raise awareness about FP symptoms and experiences and to instruct participants to form their impressions based on cues from the body and voice rather than the face. The Education + Feedback Condition added feedback about the correctness of participants’ judgments. The results showed that bias and accuracy in the two training conditions did not significantly differ, but that they were significantly less biased than controls. These findings provide preliminary evidence that such a training program can reduce negative bias towards people with FP.

### The current research

Building on this previous research, we developed a training program consisting of the same set of tips employed in Bogart & Tickle-Degnen’s study [16], but also including a brief training phase in which participants in the training condition practiced identifying emotional expressions in body language. Moreover, the training phase included an exercise in imagining experiencing emotions and expressing them with bodily cues. In addition, the current study differed from Bogart & Tickle-Degnen’s in that we implemented the program online, using Prolific Academic to recruit a broad sample of participants from the general population.

To test the efficacy of the training program, we also administered a series of tasks on which we compared the performance of those participants who had received the training (i.e. in the Trained Condition) with a separate control group (Untrained Condition). Participants in the Untrained Condition received only general information about the medical symptoms of FP (this was designed to balance the exposure to information about FP in general between the two groups), and underwent a brief practice phase in identifying emotions expressed through facial expression (this was designed to balance the amount of time spent making judgments about emotions between the two groups).

The tasks which we administered consisted of questions about brief videos of individuals with FP. In the first phase of the experiment, the questions were designed to test participants’ accuracy in identifying the emotions being expressed in the videos, as well as intensity of the emotions they attributed to the individuals in the videos, and also to probe what cues they relied upon in making these judgments. In the second phase of the experiment, participants were again presented with a video of an individual with FP describing his experiences. Now, however, they were asked questions probing how well they could attend to and recall the content of what the individual had said.

We predicted that participants in the Trained Condition would be more accurate in identifying the emotions expressed in the videos in phase 1, that they would attribute a greater intensity of emotions to the individuals in the videos, and that they would be more likely to refer to non-facial cues in indicating how they had perceived the emotions in the videos. We also predicted that in phase 2, participants in the Trained Condition would be more accurate in responding to the questions probing their ability to recall the content of what the individuals with FP had recounted in the videos.

## Materials and methods

The hypotheses, sample size, methods, exclusion criteria and planned analyses were pre-registered before data collection, and can be accessed at: http://aspredicted.org/blind.php?x=vw7ih4. All aspects of the study were carried out in accordance with the pre-registered protocol unless otherwise stated. The pre-registration specified a follow-up study testing for long-term effects of the training. However, it proved impossible to carry out this study as planned, because the privacy settings in our online experiment, for which we had ethics approval, precluded the subsequent identification of participants in each group.

### Participants

**Fig 1:**
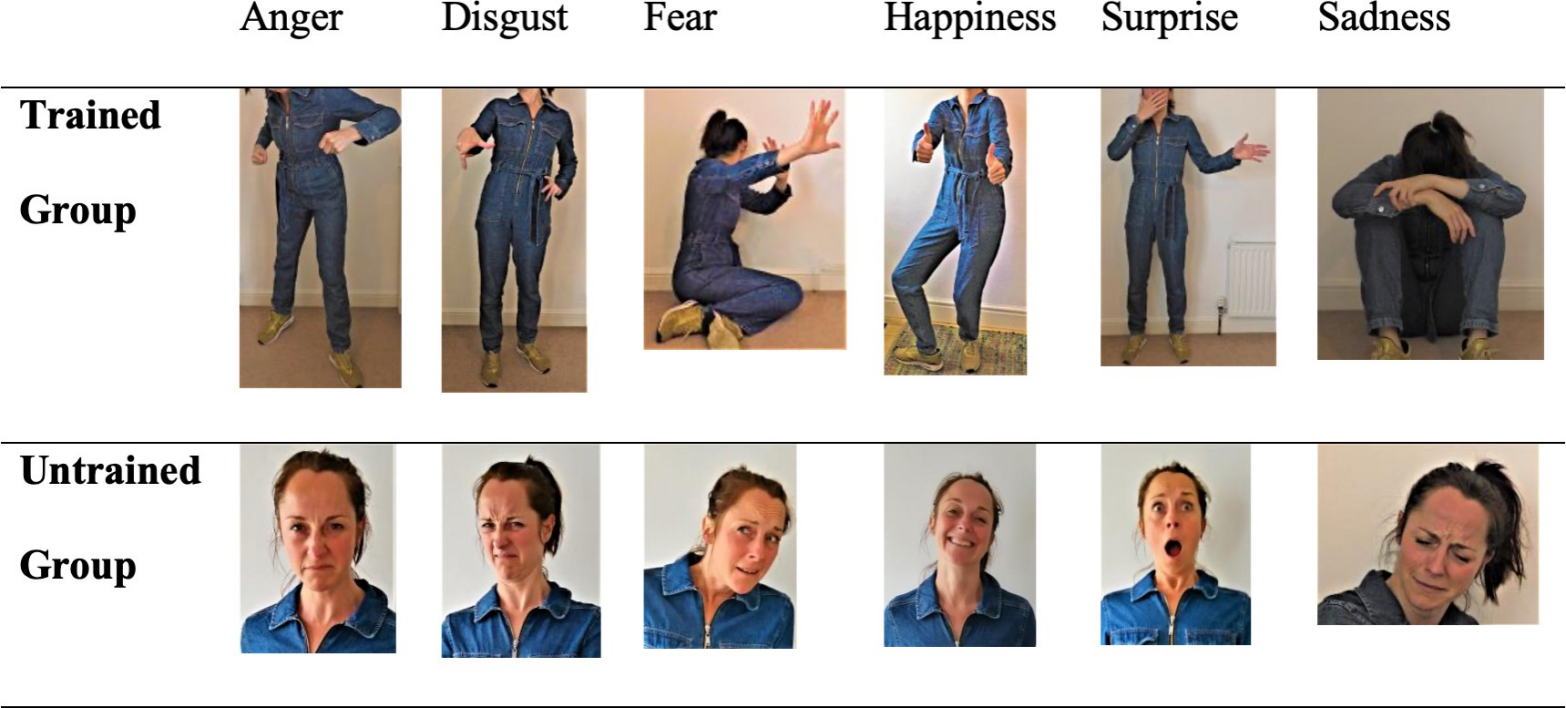
Stimuli. Differentiated Images shown to Trained and Untrained groups.

Since online experiments produce greater variability than lab-based experiments–especially when implementing a between-subjects design–we expected a high variability in our dependent variables. Moreover, we also anticipated our manipulation to have only a small effect–firstly, because our training consisted in only one brief session, and secondly, because there is less control over testing conditions in online testing. For these reasons, we opted for a large sample size (500 participants, with random allocation to the two groups). Because the online platform we used (Prolific Academic) continues recruiting new participants until the required number of participants have completed the study, a number of participants had already begun the study when the 500th participants had completed the study. Thus, 609 English-speaking adult participants were recruited via Prolific Academic to take part in the survey in English. Participants were excluded if they did not give consent, self-reported as being under 18, did not complete the experiment, failed more than one of the control questions or if the time they took to complete the survey was not within 2.5 standard deviations of the mean completion time (*M* = 1832, *SD* = 1018). After excluding 143 participants, the dataset included 466 participants (252 men, 211 women, 2 other, 1 prefer not to say). Of these, 256 participants identified their age as being between 18 to 29 (54.9%), 110 between 30 to 39 (23.6%), 51 between 40 to 49 (10.9%), 32 between 50 to 59 (6.9%) and 7 above 60 (3.6%). See S1 Table for more detailed information regarding demographics. We also note that the individual depicted in Fig 1 of this manuscript has given written informed consent (as outlined in PLOS consent form) to publish these case details, and that the two actors in the videos used in the study provided their written informed consent for their videos to be used in the study.

The experiment was conducted in accordance with the Declaration of Helsinki and was approved by the Humanities & Social Sciences Research Ethics Sub-committee (HSSREC) at the University of Warwick. Participants were paid £2 for their participation.

### Stimuli

#### Familiarisation stimuli

To create stimuli for the familiarisation phase (see below), a naive actress who did not have FP was instructed to pose using facial expressions or body movements to represent 6 targeted emotions (anger, disgust, fear, happiness, surprise and sadness). The actress had given her informed written consent. The actress’s expressions were presented as 12 separate images. Given that they were clear demonstrations of basic emotions, we expected high levels of accuracy, as we indeed observed.

See Fig 1 for 6 body images for the Trained Group and 6 facial images for the Untrained Group.

#### Test stimuli 1

For test phase 1, one female and one male with MS (both had given informed consent) were instructed to describe in their own words, events that elicited target emotions (happiness, sadness, anger). Their responses were filmed and edited into 6 thin slice clips (*M* = 20.8s) which were shown in the following order: female sadness (24s), male happiness (23s), female anger (21s), male anger (19s), female happiness (22s), male sadness (16s). See S1 File and OSF link for details about the videos.

#### Test stimuli 2

For test phase 2, we recorded a male, with MS (who had given informed consent) describing the following events in chronological order: how he first met his wife on Facebook, when they started dating, when he travelled to America to meet her family, and when they finally got married. The description contained geographic and biographical details (2.5 minutes in total).

### Procedure

After giving informed consent, participants were randomly assigned to either the Trained or Untrained Group. In both groups, participants first answered demographic questions, and then completed a familiarisation phase, followed by two test phases and a post-test phase. The full set of questions is available in S1 File. The videos are available on the OSF project page: https://osf.io/gn59u/.

#### Familiarisation phase

In the familiarisation phase, Participants in both groups read a brief general statement about the medical symptoms of Möbius Syndrome (MS) and were also provided with information about the prevalence of the condition. This ensured that participants in both groups had a similar amount of general information about MS, and also that neither group would be more motivated than the other (i.e. by having more information about the group of people most directly benefitting from the intervention).

In the Trained Group, participants were instructed not to rely on the face for information when communicating with people with FP. Next, they were asked to imagine how they would express themselves in a dating scenario if they could not use facial expressions. Participants were also informed about typical symptoms of MS including speech and facial expression difficulties which affect the movement of the eyebrows, eyes and lips. To encourage proactive thinking and highlight the importance of compensatory communication channels in those with FP, participants also answered multiple choice questions based on the content of the training session. Generic feedback was also given to participants after they answered the questions, in order to reinforce their understanding of the training. After the question and feedback, participants viewed familiarisation stimuli (6 body images) and were asked to select the correct emotion from an array of six possible emotions.

In the Untrained Group, participants were not educated about any compensatory strategies. Instead, participants were given the task of imagining how they would use facial expressions to communicate in a dating scenario. Participants were then tested on their understanding of the general statement given at the beginning of the familiarization phase in a multiple-choice format, followed by generic feedback. Finally, they were presented with familiarisation stimuli (6 facial images) and selected the emotions they deemed as best-fit to the images.

#### Test phase I

Both groups then completed Test Phase 1, in which they viewed Test Stimuli 1 in the order mentioned above. After each video, participants were asked a series of questions. Firstly, we measured participants’ ability to recognize the emotions expressed by the actor in the video, by having them selecting the correct emotion from a list of the following options: fear, anger, sadness, surprise, happiness, disgust. Secondly, to probe participants’ sensitivity to the strength of the actors’ emotional expression, irrespective of the classification of the emotion, participants were asked to rate the intensity of the perceived emotion on a five-point scale by selecting *not at all intense*, *not very intense*, *moderately intense*, *quite intense*, *or highly intense (coded as 0–4)*. Thirdly, to investigate which channels participants were conscious of using when attending to emotions, participants were asked to rate (by selecting *not at all*, *not much*, *somewhat*, *very much; coded as 0–3*) to what extent they based their decision on: body language, gesture, tone of voice, words, the actor’s face. The questions were implemented in a fixed sequence in order to ensure that participants in each group would undergo similar experiences.

#### Test phase II

Participants first watched a video of the actor describing a series of events (Test Stimuli 2), and then were presented with seven questions which probed their recollection of the content of the actor’s monologue. The full list of questions can be found in the S1 File.

#### Post-test phase

After the test phase, participants were asked several control questions in order to help us identify and exclude bots as well as participants who had not been paying attention. Finally, participants were asked to provide open-ended feedback about how useful they thought this study would be for them in interacting in the future with people with FP.

## Results

The full data set is available on the OSF project page https://osf.io/gn59u/.

### Manipulation check

To assess whether participants in the trained group understood and implemented the suggestions made in the training phase, we asked participants in both groups to report to what extent they had attended to various cues. The results showed that trained participants attended to body cues significantly more than untrained participants, *t*(464) = 3.25, *p* = .001, *d* = .3, and attended to the models’ face significantly less than untrained participants. *t*(464) = -4.72, *p* < .001, *d* = .44. However, trained participants did not attend to voice significantly more than untrained participants, *t*(464) = 1.92, *p* = .055, *d* = .18, and there was no difference between trained and untrained participants with regards to attention to words, *t*(464) = -.39, *p* = .691, *d* = .04 (see S2 Table for means and standard deviations).

### Emotion recognition accuracy

In order to investigate how the training affected participants’ ability to accurately recognize an emotion expressed by someone with FP we carried out an independent samples t-test on accuracy scores between the trained and untrained group. Accuracy scores were determined for each participant by calculating that participant’s percentage of correct responses to the six emotion recognition questions (e.g. if they got 5 out of 6 of the questions correct, their emotion accuracy score would be 0.833). This analysis did not reveal any significant difference between the two groups, *t*(464) = -.96, *p* = .343, *d* = .09.

**Fig 2:**
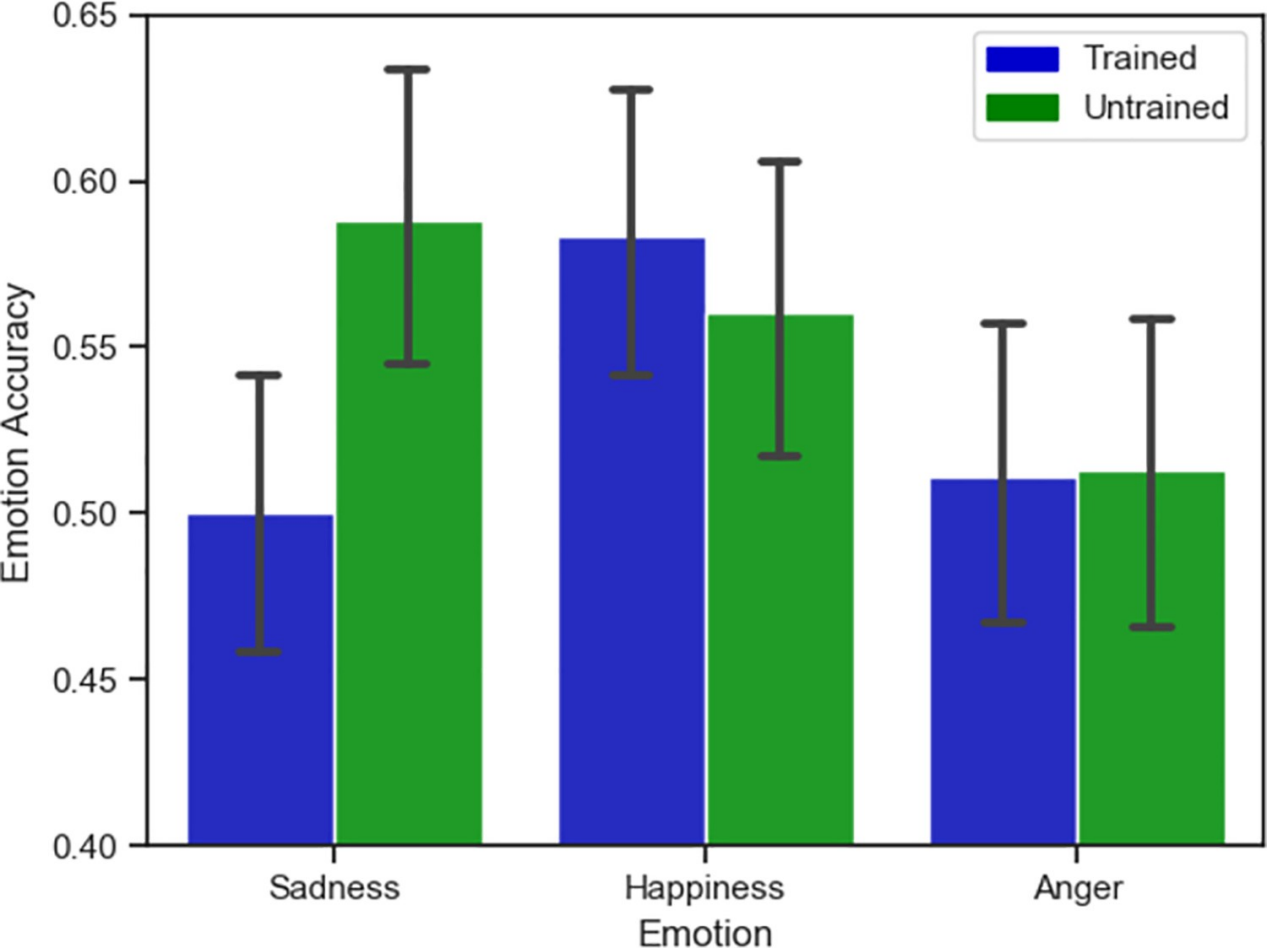
Accuracy results. Emotion Recognition Accuracy depending on Groups and Emotion Categories. Y-axis represents a truncated scale, and error bars represent 95% confidence intervals.

The extent to which the training affected participants’ ability to recognize each of the three specific emotions (sadness, happiness and anger) expressed in the videos was investigated using three independent samples t-tests. For sadness participants in the untrained group were significantly more accurate than participants in the trained group. *t*(464) = -2.62, *p* = .009, *d* = .25. However, there were no differences for happiness, *t* (464) = .59, *p* = .552, *d* = .06, or for anger, *t*(464) = -.06, *p* = .941, *d* = .006. See comparisons in Fig 2 (see S2 Table for means and standard deviations).

**Fig 3:**
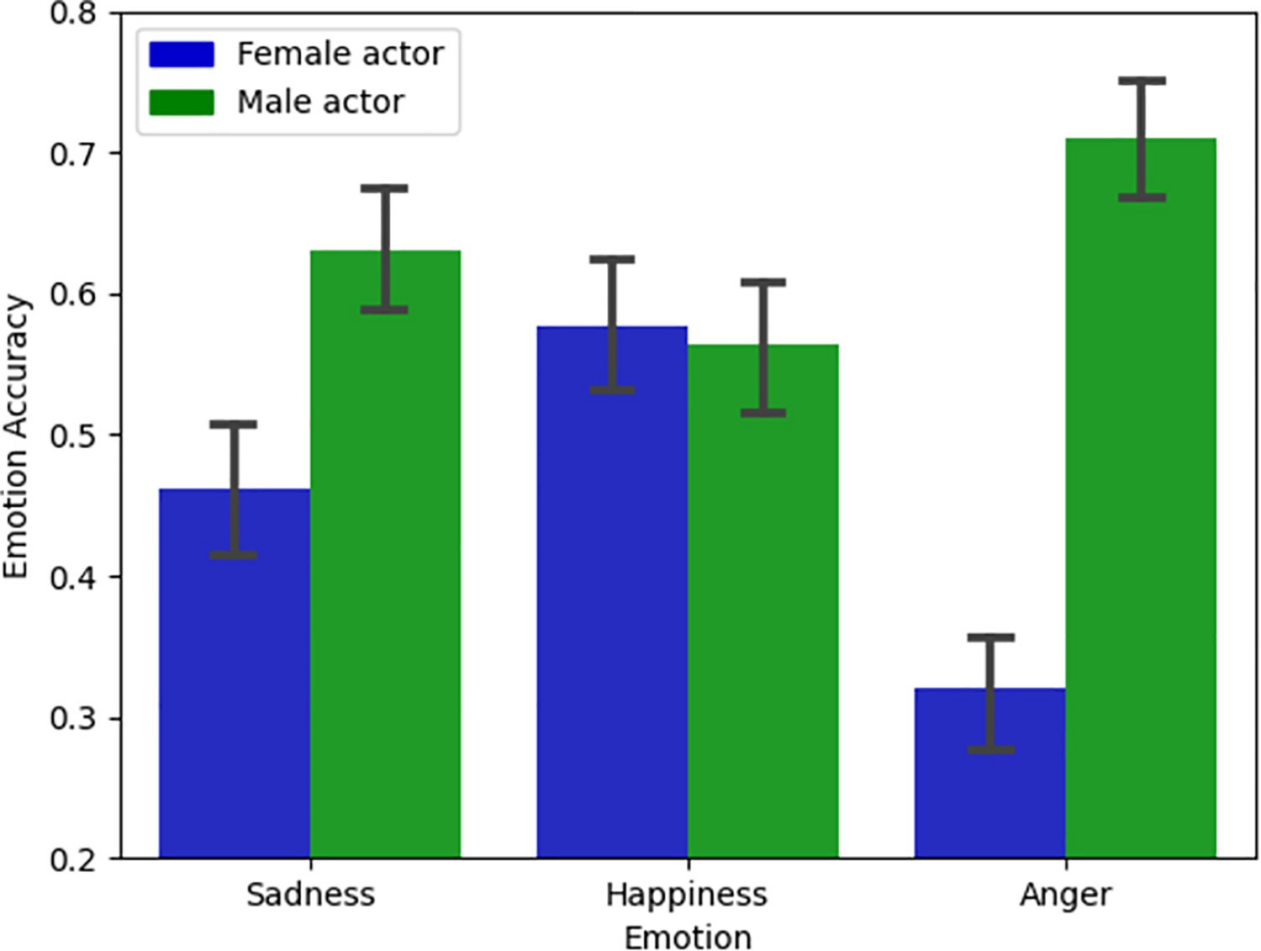
Accuracy and gender results. Emotion Recognition Accuracy depending on actor Gender and Emotion Categories. Y-axis represents a truncated scale, and error bars represent 95% confidence intervals.

In order to further investigate the extent to which the training, the emotions expressed in the videos, and the gender of the actor affected participants’ ability accurately recognize the emotion, we carried out a generalized linear mixed model (GLMM) with accuracy (correct, incorrect) as a binary dependent variable; condition (trained, untrained), emotion (sadness, happiness, anger) and actor (male, female) as independent variables. This analysis revealed a significant main effect of emotion, z = -3.93, p < .001, β = -.19, and a significant interaction between gender and emotion, z = 4.26, p < .001, β = .13 (see Fig 3), demonstrating that accurately participants could recognize each emotion depended on the gender of the actor expressing that emotion. This did not depend on whether or not the participants had received training. All other main effects, two-way interactions, and the three-way interaction were non-significant.

### Emotion intensity

We also aimed to investigate how the training affected participants’ perception of the intensity of the emotions expressed by those with FP. To gauge on average how intensely participants perceived the emotions as we took the mean of the participants emotion intensity rating (on a scale of 1–5, with 1 being ‘not-at-all intense’ and 5 being ‘highly intense’) for each of the six videos. An independent t-test revealed that those in the trained group perceived the emotions as more intense than those in the untrained group *t*(464) = 3.47, *p* < .001, *d* = .32 (see S2 Table for means and standard deviations).

**Fig 4:**
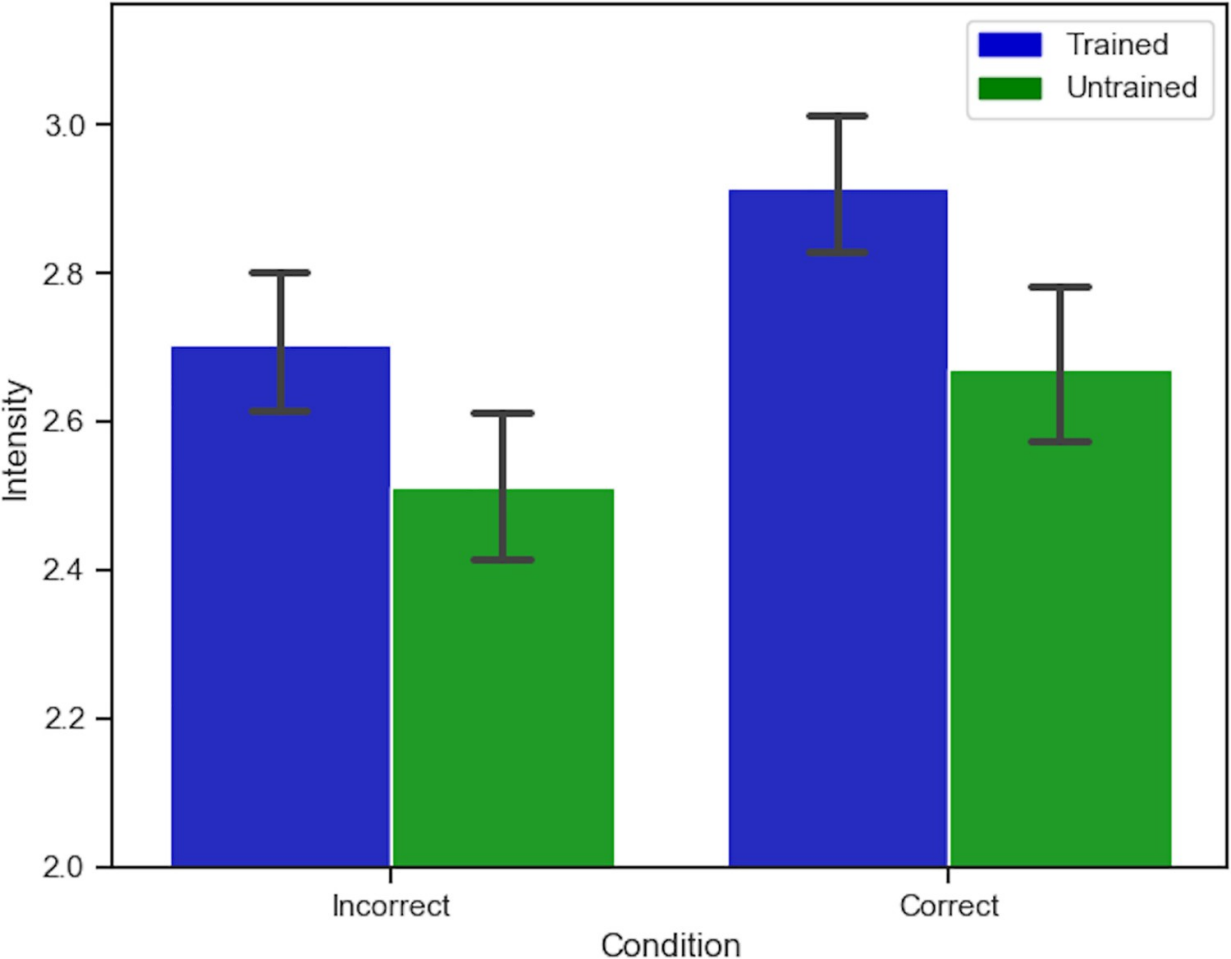
Accuracy and intensity results. Effects of Groups and Emotion Recognition Accuracy on Perceived Emotion Intensity. Y-axis represents a truncated scale, and error bars represent 95% confidence intervals.

In order to ensure that participants were not biased towards rating the wrong emotions as more intense we carried out a linear mixed model with accuracy (correct, incorrect) and condition (trained, untrained) as factors. Instead of ANOVA we chose a linear mixed model as this analysis is more robust with regards to large and unequal sample sizes. We found a significant main effect of accuracy *z* = 3.88, *p* < .001, and also of condition, *z* = 2.69, *p* = .007, but no interaction between accuracy and condition, *z* = -.71, *p* = .484. This suggests that the effect that training has on intensity is not simply down to a bias towards the wrong emotions (see results in Fig 4).

**Fig 5:**
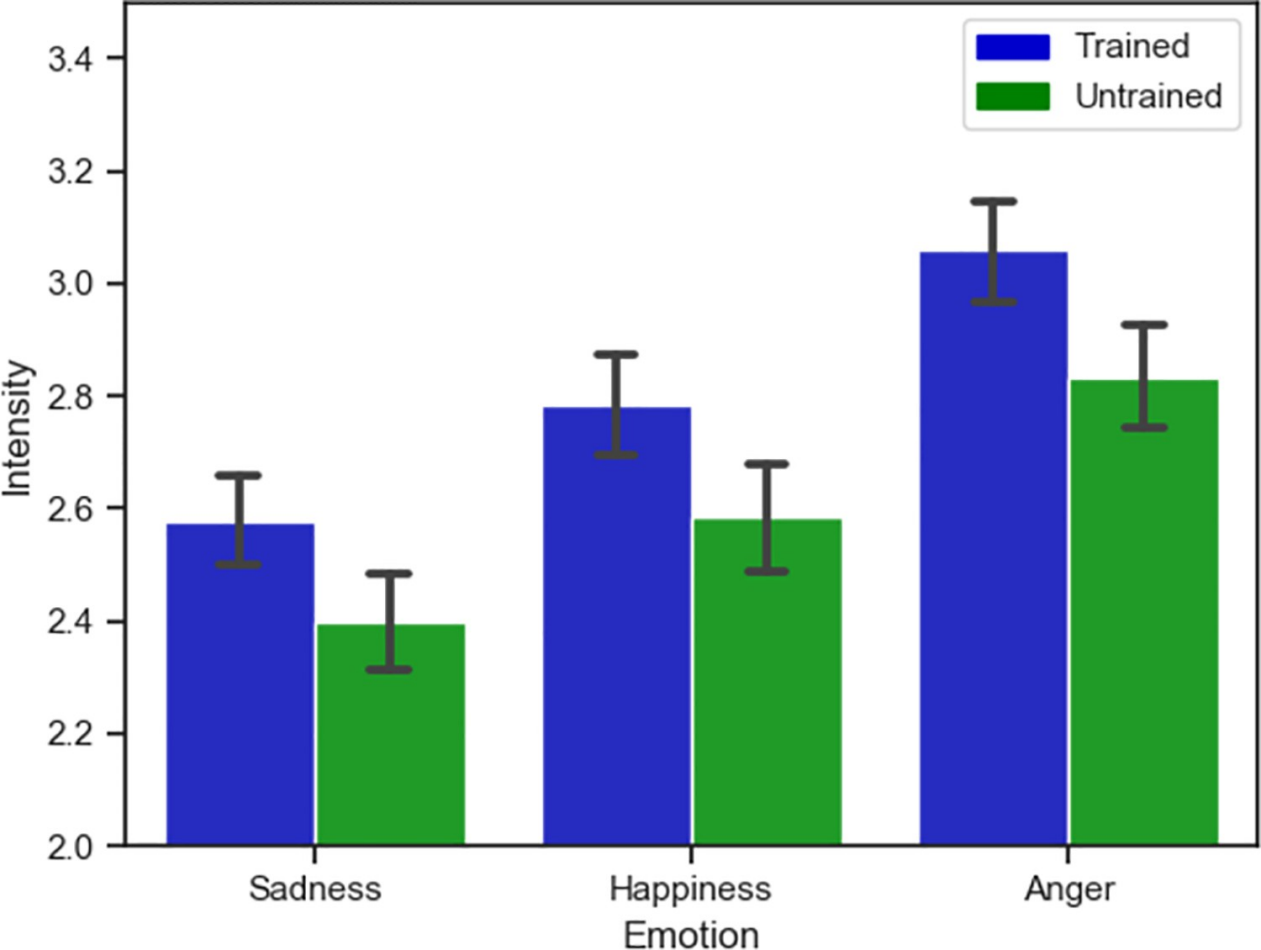
Training and intensity results. Significance of Training on Perceived Emotion Intensity. Y-axis represents a truncated scale, and error bars represent 95% confidence intervals.

Across the three emotions, separate independent t-tests showed that trained participants rated emotions as more intense than untrained participants, for sadness, *t*(464) = 2.86, *p* = .007, *d* = .25; for happiness, *t*(464) = 2.61, *p* = .009, *d* = .24; and for anger, *t*(464) = 3.02, *p* = .002, *d* = .28. See Fig 5 for the results.

In order to further investigate the extent to which the training, the emotions expressed in the videos, and the gender of the actor affected participants’ ability to accurately recognize the emotion, we carried out a LMM: with emotion intensity (1–5) as a dependent variable, and condition (trained, untrained), emotion (sadness, happiness, anger) and actor (male, female) as independent variables. This analysis revealed a main effect of condition, z = -3.61, p < .001, β = -.21, a main effect of emotion, z = 2.47, p < .014, β = .15, and a main effect of gender, z = -6.29 p < .001, β = -.3. All two-way and three-way interactions were not significant.

### Factual recognition

As well as recognizing emotions, another aim of this study was to investigate how the training affected participants’ ability to derive factual information from those with FP. We took participants’ overall accuracy for the seven questions (% correct) and conducted an independent samples t-test, *t*(464) = .12, p = .911, *d* = .01, which revealed no differences between the trained and untrained group (see S2 Table for means and standard deviations).

### Exploratory analyses

We also ran a battery of exploratory analyses on the data from this study. The first set of analyses aimed to investigate to what extent participants rated (on a scale of 1–4 with 1 being ‘not at all’ and 4 being ‘very much’) relying on different cues to emotion (face, words, voice, gestures, and body language), and whether participants’ responses regarding emotion (mean emotion accuracy and mean emotion intensity) and facts (mean factual accuracy) depended which of these cues they attended to. For each cue, we took the participants’ mean rating for each of the six emotion expression videos, in order to tell us on average how much each participant relied on each cue when attending to the actor in the videos. Because gesture and body language ratings were highly correlated (*r* = .77), we collapsed them into one variable named bodily cues.

We then correlated participants’ mean emotion accuracy, emotion intensity, and factual accuracy with the mean score for each of the ratings (indicating their overall tendency to attend to each of these cues).

Emotion accuracy was moderately positively correlated with attention to the models’ words, *r*(465) = .45, *p* < .001, and moderately negatively correlated with attention to the models’ face *r*(465) = -.31, *p* < .001. Moreover, emotion intensity ratings were moderately positively correlated with attention to bodily cues, *r*(465) = .38, *p* < .001, and voice, *r*(464) = .4, *p* < .001. There were also weak correlations for words, *r*(465) = .23, *p* < .001 and face, *r*(464) = .21, *p*< .001. Finally, factual accuracy was moderately positively correlated to attention to the models’ words, *r*(465) = .39, *p* < .001, and moderately negatively correlated with attention to the models’ face, *r*(465) = -.28, *p* < .001.

We used the Natural Language Toolkit (NLTK), a python based Natural Language Processing (NLP) toolbox in order to explore the optional open-ended responses that participants gave at the end of the study (378 out of 466 participants chose to give open ended responses), in order to get an idea of how the training affected how participants felt about the task. NLTK is powered by pre-existing libraries and corpora which allow for text processing, text classification, and semantic interpretation. Before we carried out any analysis, we carried out several steps of pre-processing. Firstly, we split each participant sentence into single words (tokenization), removed any stopwords (words with no semantic content) and then transformed the remaining words into their respective root word (lemmatization). This process left us with 195 sentences (104 from the trained group and 91 from the untrained group).

**Fig 6:**
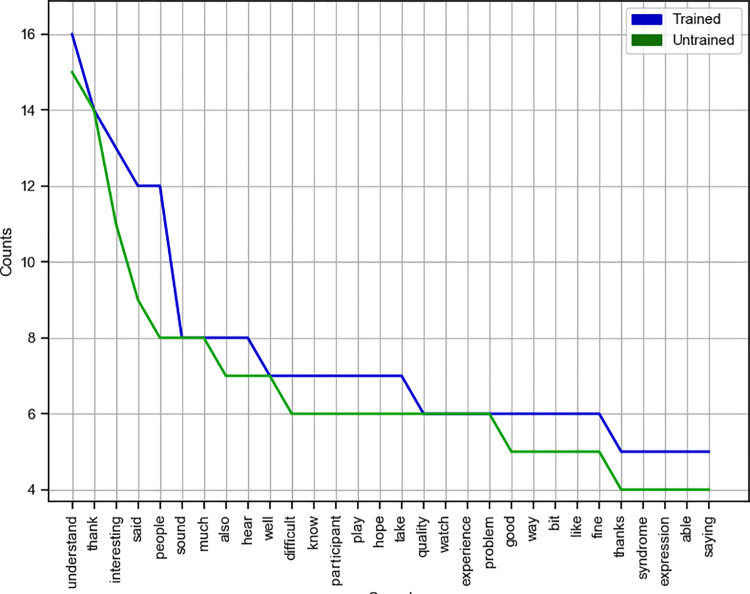
Themes. Frequency Distribution of Common Themes based on Participants’ Feedback.

Firstly, we plotted a frequency distribution for each group in order to identify common themes in the participants sentences. See Fig 6.

Secondly, we carried out a sentiment analysis on each of the participants’ pre-processed responses, in order to get an idea of how participants felt after the task. Each response was first assigned a score (sentence polarity) from -1 (negative) to +1 (positive) on the basis of the valence of the words in that sentence (this was done automatically based on corpora provided by NLTK). For example, within a sentence, positively valanced words such as ‘interesting’, ‘thank’ and ‘hope’ would increase the sentence polarity score, and negatively valanced words such as ‘difficult’ and ‘problem’ would decrease the sentence polarity score.

This gave us a crude measure of how positively or negatively participants felt towards the task. Interestingly, an independent samples t-test comparing the sentiment scores between the two groups was significant *t*(190) = 2.78, *p* = .006, *d* = .4, demonstrating that trained participants responses (M = .26, SD = .36) were significantly more positive than untrained participants responses,(M = .11 SD = .37), with regards to the sentiment of their open ended responses.

## Discussion

We tested the efficacy of a web-based training program which encouraged participants (in the Training Group) to attend to the gestures, body language and speech of individuals with FP rather than to the face. Contrary to our hypothesis, the results from both studies showed that participants in the Trained group were not more accurate at emotion recognition than participants in the Untrained group. In fact, participants in the Untrained group were more accurate than participants in the Trained group at recognizing sadness. This is surprising insofar as we expected the training to reduce the tendency to perceiving people with FP as sad [16] and thus to increase accuracy. It is worth noting that participants in both conditions exhibited a high level of accuracy– 50–60%, which was well above chance (~16.67%)–at emotion recognition in both conditions. It is therefore possible that our results did not reveal differences in emotion recognition because the control group was already at ceiling in their accuracy, rather than because the training for the treatment group was ineffective. A further possibility is that the training did not improve accuracy in the test phase because the emotions expressed in the test phase were subtler and/or more complex, whereas the emotions depicted in the training phase were simple and stylised.

Additionally, we found that accuracy differed depending on the type of emotion being expressed, and the gender of the model expressing that emotion. Moreover, an interaction between gender and type of emotion suggests that people were better at picking up on particular emotions from the male actor than from the female actor. Because we only had two models, we cannot rule out the possibility that other differences between the two actors (other than gender) may also explain this effect. Further research should investigate how prior conceptions of those with FP differ depending on their gender and explore how a better understanding of these prior conceptions can be used to optimize training and outreach programmes.

The results for perceiving emotional intensity, however, do support our hypothesis: participants in the Trained group in general perceived the individuals in the videos as exhibiting a higher degree of emotional intensity. This suggests that, though the training did not improve their ability to categorise perceived emotions, it may have made them more sensitive to emotional expression per se—perhaps by reducing a bias to perceive people with FP as emotionally flat or disinterested [16]. The extent to which the training affected the perception of emotional intensity also differed depending on the emotion that was being expressed as well as the gender of the actor expressing that emotion. However, these factors did not affect the extent to which the training increased ratings of emotional intensity (i.e. these factors did not interact with the condition), suggesting that the training was equally effective in increasing participant’s perception of the intensity of the emotions of those with FP, regardless of who was expressing the emotion, or what they were expressing. It is worth noting, however, that we did not ask the actors with FP to rate the intensity of their own emotions, and that we have no objective measure of intensity. As a result, it is not possible to ascertain whether observers became more accurate in assessing emotion intensity or whether the training introduced a bias towards perceiving emotions as more intense.

For Phase 2, we predicted that the training would facilitate participants’ ability to pay attention to and recall the content of what the individual in the video was recounting. The results, however, show no significant difference between conditions. It may be that participants who have just received the training are impaired by the cognitive load arising from the need to remember and apply what they have just learned. If so, then we might expect that people would perform better if they were prevented from viewing the faces of the individuals in the videos (e.g. if the faces were blocked) than if instructed to deliberately avoid looking at the faces. Indeed, we should expect that blocking out the faces of individuals with FP in videos would improve observers’ accuracy, whereas blocking out the faces of individuals without FP would impair observers’ accuracy.

The exploratory analyses revealed several interesting findings. Firstly, we observed that emotional accuracy was moderately positively correlated with attention to the models’ words, and moderately negatively correlated with attention to the models’ face. This is consistent with what our hypothesis should lead us to expect: paying more attention to what a person with FP is saying and less attention to their face is beneficial with respect to accurately perceiving their emotional state. Secondly, participants who paid more attention to bodily cues and to the tone of voice perceived a higher degree of emotional intensity than participants who paid less attention to these sources of information. Again, this is what our hypothesis would lead us to expect. Thirdly, accuracy in recalling what the individual had said was moderately positively correlated to attention to the words spoken and moderately negatively correlated with attention to the face. This, too, is consistent with our hypothesis. The results of these exploratory analyses suggest that encouraging people to attend to bodily cues and speech (content and tone of voice) may indeed improve accuracy at emotion recognition and help people to pay attention to and recall speech content, but that our training may not have been sufficiently extensive to produce clear effects.

Finally, exploratory analyses also revealed that participants in the Trained Group used more positively valenced words in the open-ended feedback questions at the end. We may speculate that this reflects either a more positive impression of the people in the videos they had viewed, a more positive experience viewing the videos, or both.

It must be acknowledged that the current study had a number of limitations, which future research may improve upon. For example, there was no long-term measure of the effectiveness of the intervention. Future research could administer a follow-up probe several weeks or months later in order to gauge any long-term effects of the training. Moreover, in the current study, no information was gathered about participant’s prior experience of interacting with individuals with FP or other conditions that might affect their performance on the tasks administered here. In addition, our training focused on bodily cues but did not include exercises in attending to speech; future training programs could include a component addressing speech (content and tone of voice). A further limitation pertains to the study design: in the control condition, participants were presented with images of facial expressions, which may have enhanced any pre-existing tendency to preferentially attend to the face rather than to gestures, prosody or other cues. It would be valuable for future research to develop an alternative control condition that is free of this limitation. Finally, the range of emotions used for the current study was quite restricted: only three basic emotions were used in the current study. And indeed, in phase 2 of the experiment, only positive emotions were implemented. It would be valuable for future research to implement a broader range of emotions and to investigate whether more complex or subtle emotions might elicit a difference between trained and untrained groups in recognition accuracy.

In sum, building on the results of previous research [15], our findings support the hypothesis that even brief training in attending to non-facial cues when interacting with individuals with FP may improve social perception and reduce bias. It would be valuable for future research to develop more extensive, interactive training programs for medical professionals, educators, and others who are likely to interact with individuals with FP.

## Supporting information

S1 File Procedural details. Details of each phase of the procedure for both groups.
(DOCX)

S1 Table Demographics. Breakdown of demographic information and time to complete survey across both conditions.
(DOCX)

S2 Table Descriptives. Means (and standard deviations) of the main dependent variables tested in our study.
(DOCX)
