# Supplementary material for: Web-based sensitivity training for interacting with facial paralysis
Source: PLoS One. 2022 Jan 21;17(1):e0261157. doi: 10.1371/journal.pone.0261157 (PMC8782395; doi:10.1371/journal.pone.0261157)
Supplement: S1 File — Details of each phase of the procedure for both groups. (DOCX) [file pone.0261157.s001.docx]

**S1 File. Procedural details**

**Demographic questions for both groups**

1.     What is your age?

· Under 18

· 18 to 29

· 30 to 39

· 40 to 49

· 50 to 59

· 60 to 69

· 70 and above

2.     What is your gender?

· Male

· Female

· Other ____

· Prefer not to say

3.     What is your ethnicity?

· Caucasian

· Middle Eastern

· Latino/Hispanic

· African

· Caribbean

· South Asian

· East Asian

· Mixed

· Other

4.     Which of the following best describes your current occupation?

· Unemployed

· Health care or social assistance

· Business / Human Resources

· Education

· Arts, entertainment or recreation

· Other ____

**Familiarisation trained group**

1.     Are you aware of Moebius Syndrome?

· No

· Yes

2.     What do you know about Moebius Syndrome? (This question only applied if the answer was ‘yes’ to question 1 above.)

3.     What is Moebius Syndrome?

Moebius Syndrome is a rare disorder affecting approximately only 200 people in the UK.  Moebius syndrome is congenital, meaning it is present at birth; it does not get worse or better over time.

Most people born with Moebius Syndrome experience facial paralysis (usually on both sides) as a result of absent or underdeveloped cranial nerves from birth. The exact cause of this condition is unknown, but it may be due to a problem with blood flow in the womb.

There are a number of other symptoms that may be present, such as inability to move the eyes side-to-side, inability to blink, crossed eyes, speech problems, cleft palate, jaw/dental abnormalities, respiratory problems, limb abnormalities, and occasionally cognitive disabilities.

How do people with Moebius Syndrome feel in general?

People with Moebius Syndrome often cite society’s lack of awareness as their primary concern. People they interact with may notice that they look different but don’t know about facial paralysis.

Having facial paralysis can make social interaction difficult because people are accustomed to receiving feedback from a person’s facial expressions. But many people with Moebius syndrome find other ways to express themselves.

4.     Exercise:

Imagine that you couldn't smile, frown, or raise your eyebrows, and that you had difficulties speaking because of lip paralysis. And imagine that you were on a first or second date with someone and wanted to indicate that you liked them.

Please describe how you might express this using body language, hand gestures and tone of voice:______________

5.     These are the typical symptoms of Moebius Syndrome:

· No/asymmetrical facial expression – for example with an inability to smile, frown, raise eyebrows, puckering of lips;

· Speech difficulty as a result of paralysed lips;

· Inability/difficulty blinking resulting in dry eyes;

· Inability to follow objects moving across horizontally (also known as tracking) without moving their head.

6.     When interacting with people who have facial paralysis, it can be difficult to read their emotions from their facial expressions, since their faces are paralysed. It can also be difficult to understand their speech, because they lack control of their lips. To try to understand them better, which of the following option(s) do you think may / may not be useful to pay attention to? (Participants were asked to categorise the following options either as ‘useful to pay attention to’ or as ‘NOT very useful to pay attention to’):

· Body language

· Gesture

· Tone of voice

· Words

· Their faces

7.     Good job!  When interacting with others with facial paralysis, please remember:

· It is NOT very useful to pay attention to their faces or the extent to which their faces are expressionless, asymmetrical, or saggy, because these are simply symptoms of facial paralysis.

· It is more useful to pay attention to their **body language, gestures, tone of voice, words.**

8.     What emotion is the body language expressing below? (The question applied to each of the six images which were presented sequentially.)

·   Fear

·   Anger

·   Sadness

·   Surprise

·   Happiness

·   Disgust

**Familiarisation control group**

1.     Same as in Trained Group

2.     Same as in Trained Group

3.     Same as in Trained Group

4.     Exercise:

Imagine that you were on a first or second date with someone and wanted to indicate that you like them.

Please describe the facial expressions that you might use:_____

5.     Same as in Trained Group

6.     What are the symptoms of Moebius Syndrome? Please drag your answer(s) to the right boxes. (Participants were asked to categorise the following options either as ‘useful to pay attention to’ or as ‘NOT very useful to pay attention to’):

· Speech Difficulty

· Inability to blink & smile

· Inability to frown

· Inability to raise the eyebrows

· High blood pressure

7.     Good job!  These are all symptoms of Moebius Syndrome apart from high blood pressure.

8.     What emotion is the body language expressing below? (The question applied to each of the six images which were presented sequentially.)

·   Fear

·   Anger

·   Sadness

·   Surprise

·   Happiness

·   Disgust

**Test phase I for both groups**

Participants viewed six videos and were required to watch them once only.

Video 1 (sad) lasted 24 seconds; it showed a woman with MS talking about being alone at home.

Videos 2 (happy) lasted 23 seconds; it depicted a man with MS describing his happy feeling when he first met his wife.

Video 3 (angry) video lasted 21 seconds; it showed a woman with MS commenting on difficulties with her partner.

Video 4 (angry) lasted 19 seconds; it showed a man with MS describing his anger while in Video 5 (happy) lasted 22 seconds; it depicted a woman with MS expressing her happiness spending time with her extended family.

Video 6 (sad) lasted 16 seconds; it showed a man with MS talking about feeling sad when people didn’t understand his speech.

The following questions applied to each of the videos:

1.     What emotion was the woman experiencing during the event as described in the video?

·       Fear

·       Anger

·       Sadness

·       Surprise

·       Happiness

·       Disgust

2.     How would you rate the intensity of her/his emotion?

·       Not at all intense

·       Not very intense

·       Moderately intense

·       Quite intense

·       Highly intense

3.     To what extent did you base your answer on these different aspects?

·       Body language (Not at all; Not very much; Somewhat; Very much)

·       Gesture (Not at all; Not very much; Somewhat; Very much)

·       Tone of voice (Not at all; Not very much; Somewhat; Very much)

·       Words (Not at all; Not very much; Somewhat; Very much)

·       Face (Not at all; Not very much; Somewhat; Very much)

**Test phase II for both groups**

Participants watched a video and were asked the following questions.

1.     What did participant one think about participant two’s family?

·       that they were amazing.

·       that they were hard-working.

·       that they were intelligent.

·       that it was a big family.

·       I don't know.

2.     What did participant two’s dad suggest to participant one?

·       that participant one go to the US.

·       that participant two go to the UK.

·       that He and participant one go to California together.

·       that participant 1 could Skype with him.

·       I don't know.

3.     Why did participant two’s dad ask participant one to return to the US?

·       to surprise participant two.

·       to help on the farm.

·       to see his friends and extended family.

·       to go to a conference.

·       I don't know.

4.     What did participant one do in the US?

·       He proposed to participant two.

·       He looked for a job.

·       He went to a conference in New York.

·       He helped participant two’s dad on the farm.

·       I don't know.

5.     How often had participant one been to California?

·       A few times

·       Once

·       Often

·       Never

·       I don't know.

6.     Which holiday did participant one and participant two spend together after he arrived in the US?

·       Valentine's Day

·       Halloween

·       Christmas

·       Easter

·       I don't know.

7.     How did participant two’s parents feel about their relationship?

·       Supportive

·       Indifferent

·       They didn't know about it.

·       Not supportive

·       I don't know.

8.     Where did they put their wedding footage?

·       Facebook

·       Instagram

·       Youtube

·       Reddit

·       I don't know.

**Control questions for both groups**

1. Was the sound turned on during the survey?

- Yes
- No

1. What was in the videos you watched?

- People talking about their experience.
- Animal foraging for food in the forest.
- Children learning in a classroom.
- Seaside scenery

1. How many dogs did you see in the videos?

- None
- One
- Two
- Three

1. In the videos, where were the people sitting?

- In a room
- At the seaside
- In a forest
- In a restaurant
