# Supplementary material for: Web-based sensitivity training for interacting with facial paralysis
Source: PLoS One. 2022 Jan 21;17(1):e0261157. doi: 10.1371/journal.pone.0261157 (PMC8782395; doi:10.1371/journal.pone.0261157)
Supplement: S1 Table — Breakdown of demographic information and time to complete survey across both conditions. (DOCX) [file pone.0261157.s002.docx]

**S1 Table**

Demographics. Breakdown of demographic information and time to complete survey across both conditions.
