# Supplementary material for: Web-based sensitivity training for interacting with facial paralysis
Source: PLoS One. 2022 Jan 21;17(1):e0261157. doi: 10.1371/journal.pone.0261157 (PMC8782395; doi:10.1371/journal.pone.0261157)
Supplement: S2 Table — Means (and standard deviations) of the main dependent variables tested in our study. (DOCX) [file pone.0261157.s003.docx]

**S2 Table**

Descriptives. Means (and standard deviations) of the main dependent variables tested in our study.
